# Down‐regulation of RCC1 sensitizes immunotherapy by up‐regulating PD‐L1 via p27^kip1^/CDK4 axis in non‐small cell lung cancer

**DOI:** 10.1111/jcmm.16383

**Published:** 2021-02-25

**Authors:** Xiaozhu Zeng, Maoxi Zhong, Yumeng Yang, Zhi Wang, Yuxi Zhu

**Affiliations:** ^1^ Department of Oncology The First Affiliated Hospital of Chongqing Medical University Chongqing China; ^2^ Department of Oncology Jinshan Hospital of the First Affiliated Hospital of Chongqing Medical University Chongqing China; ^3^ Chongqing Clinical Cancer Research Center Chongqing China

**Keywords:** ICI, non‐small cell lung cancer, p27^KIP1^/CDK4, PD‐L1, RCC1

## Abstract

In recent years, although Immune Checkpoint Inhibitors (ICIs) significantly improves survival both in local advanced stage and advanced stage of non‐small cell lung cancer (NSCLC), the objective response rate of ICI monotherapy is still only about 20%. Thus, to identify the mechanisms of ICI resistance is critical to increase the efficacy of ICI treatments. By bioinformatics analysis, we found that the expression of regulator of chromosome condensation 1 (RCC1) in lung adenocarcinoma was significantly higher than that in normal lung tissue in TCGA and Oncomine databases. The survival analysis showed that high expression RCC1 was associated with the poor prognosis of NSCLC. And the expression of RCC1 was inversely related to the number of immune cell infiltration. In vitro, knockdown of RCC1 not only significantly inhibited the proliferation of lung adenocarcinoma cells but also increased the expression levels of p27^kip1^ and PD‐L1, and decreased the expression level of CDK4 and p‐Rb. In vivo, knockdown of RCC1 significantly slowed down the growth rate of tumour, and further reduced the volume and weight of tumour model after treated by PD‐L1 monoclonal antibody. Therefore, RCC1 could up‐regulate the expression level of PD‐L1 by regulating p27^kip1^/CDK4 pathway and decrease the resistance to ICIs. And this study might provide a new way to increase the efficacy of PD‐L1 monoclonal antibody by inhibiting RCC1.

## 1 INTRODUCTION

Lung cancer is one of the leading malignant tumour that seriously endangers human health across nations and races.^1^ The prognosis of advanced lung cancer is still poor, although the development of chemoradiotherapy, targeted therapy and immunotherapy have significantly changed the clinical practice in the past 30 years.^2^, ^3^ And the overall response rate of ICI is only around 20% for lung cancer patients. It has been found that the expression level of PD‐L1 is one of the factors might predict the efficacy of ICI.^4^, ^5^, ^6^ Therefore, the factors that affect the expression of PD‐L1 deserve further study.

Regulator of chromosome condensation 1 (RCC1) is a guanine nucleotide exchange factor on Ras‐related nuclear (RAN) protein. RCC1 catalyses the RanGDP to RanGTP which might participate in the proliferation, invasion and metastasis of tumour. Moreover, RCC1 could regulate some exchange of factors between cytoplasm and nucleus, and influence the signal transduction and downstream molecules in cells.^7^, ^8^

p27^kip1^ is a cyclin‐dependent kinase inhibitor which could regulate the cell cycle. It is highly expressed in G0 and early G1 phase to inhibit cell proliferation, thus disturbed a series of cell functions.^9^ RCC1 might promote the transport of p27^kip1^ from nucleus to cytoplasm through RanGTP, resulting in the decomposition and inactivation of p27^kip1^ in cytoplasm.

Cyclins could combine with its cognate cyclin‐dependent kinase (CDK), and the periodic synthesis and degradation of cyclins regulate the progress of each cell cycle. Among them, Cyclin D forms a complex with CDK4 (cyclin D‐CDK4) to activate the function of CDK4 and promote cell cycle progress from G1 phase to S phase.^10^ However, overexpression of CDK4 could strongly phosphorylate Retinoblastoma protein (p‐Rb) to promote cell from G1 to S phase.^11^ On the other hand, p27^kip1^ is a CDK inhibitor of KIP/CIP family, which could inhibit the activity of cyclin CDK complex in G1/S phase.^12^, ^13^, ^14^ It was reported cyclin D‐CDK4 complex contributed to regulate the expression level of programmed cell death‐ligand (PD‐L1). And CDK4 inhibitors increase the expression of PD‐L1 largely through inhibiting the phosphorylation of SPOP and promote the degradation of SPOP by APC/CCdh1.^15^, ^16^

Therefore, we intended to elucidate the effect of RCC1 on the expression of PD‐L1 by regulating p27^kip1^/CDK4 pathway, and on the growth, cell cycle, resistance to PD‐L1 inhibitor both in vitro and in vivo.

## 2 MATERIALS AND METHODS

### 2.1 Bioinformatics analysis and verification

The RNASeq data for human cancers were downloaded from The Cancer Genome Atlas (TCGA) databases (https://cancergenome.nih.gov/). The expression level of RCC1 gene in LUAD and LUSC was identified from TCGA and Oncomine database (https://www.oncomine.org/resource/login.html). The difference of mRNA expression between cancer tissue and normal tissue was obtained as the parameters of *P*‐value <.05, fold change >2, and gene ranking in the top 10%. Immunohistochemistry (IHC) datasets (The Human Protein Atlas database) was used for further validation of our results.

### 2.2 Kaplan‐Meier plotter

The Kaplan‐Meier plotter (http://kmplot.com/analysis/) is an online database, which could assess the effect of 54 000 genes on survival in 21 types of cancer. We used this platform to evaluate the prognostic value of RCC1 expression in patients with lung adenocarcinoma or squamous cell carcinoma.

### 2.3 Prognoscan

Prognoscan is a useful online tool for assessing the association between specific gene expression and prognosis of cancer patients and based on a large collection of cancer microarray datasets with clinical annotation. We used the Prognoscan platform to validate the prognostic value of RCC1 expression in patients with lung adenocarcinoma or squamous cell carcinoma.

### 2.4 TISIDB analysis

The TISIDB database (http://cis.hku.hk/TISIDB) integrates 4176 records from 2530 publications and reported 988 genes related to anti‐tumour immunity. It's a web portal for tumour and immune system interaction, which integrates multiple heterogeneous data types.^17^ We used TISIDB database to estimate the survival of RCC1 and relationship between RCC1 and immune cells.

### 2.5 Cell culture

Human lung cancer cell lines (A549, H1299) were purchased from ATCC (American Type Culture Collection, USA). Mouse lung cancer line (Lewis lung carcinoma, LLC) was obtained from The University of Texas MD Anderson Cancer Center (USA). A549 and H1299 cells were maintained in RPMI 1640 (Biological Industries, Israel) supplemented with 10% foetal bovine serum (Biological Industries, Israel), penicillin (100 U/mL; Beyotime, China) and streptomycin (10 mg/mL; Beyotime, China). LLC cells were maintained in high glucose‐DMEM (4.5 g/L d‐Glucose; Biological Industries, Israel) supplemented with 10% foetal bovine serum (Biological Industries, Israel), 100 U/mL penicillin and 10 mg/mL streptomycin. The cells were maintained in an incubator in an atmosphere of 37°C with 5% CO_2_.

### 2.6 SiRNAs and transfection

We seeded cells in 6 cm dishes at 1 × 10^5^ cells concentration and cultured it in RPMI 1640 supplemented with medium for 24 hours. Small interfering RNAs (siRNAs) targeting RCC1 and negative control siRNAs were purchased from GenePharma (GenePharma, Shanghai, China), and transfected into cells using RFect siRNA transfection reagent (Bio‐trans, Jiangsu, China) following the manufacturer's instructions. The siRNA sequences were as follows: siRCC1‐1, 5′‐CACCGUGUGUCUAAGCAAATT‐3′; siRCC1‐2, 5′‐GGCCAGCAUACAGUCUUAUTT‐3′. Four hours after transfection, the culture medium was replaced with 1640 medium plus 10% foetal bovine serum.

### 2.7 Quantitative Reverse‐Transcription PCR (qPCR)

Forty‐eight hours after transfection, we harvested cells to analyse the effect of mRNA knockdown by qPCR. RNA extraction was carried out using the Trizol Reagent (Invitrogen, USA) according to the manufacturer's instructions. According to manufacturer's instructions, cDNA was synthesized using random primers with the PrimeScript™ RT Reagent Kit with gDNA Eraser (Takara, Biotechnology, Japan). Reverse‐transcription was performed according to the instruction (Takara, Biotechnology, Japan). The qPCR reaction was performed using an SYBR Green PCR kit (Takara, Biotechnology, Japan) with primers (Table S1) on a Real‐Time PCR system (Thermo scientific, USA). PCR was performed under the following conditions: 95°C for 3 minutes, followed by 45 cycles of 95°C for 10 seconds and 58°C for 30 seconds. The relative mRNA levels were determined by the Cycler threshold method, with GAPDH serving as the housekeeping gene.

### 2.8 Western blot

After 72 hours of transfection, cells were harvested and lysed in RIPA buffer mixed with 1% protease inhibitor mixture at 4°C for 30 minutes. The protein concentration was determined by the BCA method. The protein samples (40 μg) were divided by SDS‐PAGE and electrotransferred to PVDF membranes. Next, the membranes were blocked with 5% non‐fat milk for 2 hours and incubated overnight at 4°C with primary antibodies: RCC1 (1:1000, Abcam, USA), p27^kip1^ (1:1000, Abcam, USA), CDK4 (1:1000, ABclonal, China), PD‐L1 (1:1000, Abcam, USA), p‐Rb^Ser780^ (1:1000, Abways, China) and GAPDH (1:1000, Abcam, USA). Then, we washed the membranes with TBST buffer three times and further incubated with secondary antibodies (1:10 000, Immunoway, USA) for 1 hours at 25°C. Finally, the membranes were washed three times by TBST and freshly prepared ECL fluid (Beyotime Institute of Biotechnology, China) was applied for colour development in a dark room. The grey intensity analysis of western blotting images was carried out by ImageJ software.

### 2.9 CCK‐8

The transfected cells (2 × 10^3^/well) were placed in 96‐well plates with 200 μL medium per well for 12 hours, 24 hours, 48 hours, 72 hours and 96 hours, the fresh medium was mixed with CCK8 reagent (Dojindo, China) in a ratio of 100:1. Removed the original medium and added 100 μL mixture in each hole, then incubated for 2 hours. The absorbance at 450 nm was demonstrated by microplate reader (Bio‐Rad Laboratories, Inc).

### 2.10 Flow cytometry analysis of cell cycle and cell apoptosis

To analyse the cell cycle, cells were transfected with RCC1‐siRNA and NC‐siRNA, and digested and washed twice with PBS and fixed with 75% precooled ethanol. Subsequently, the cell cycle analysis was performed by flow cytometry (BD Biosciences, USA).

For cell cycle apoptosis, after the same transfection for 48 hours, the cells were collected with PBS and centrifuged 3 times. Apoptosis was then detected by Annexin V‐FITC APOPTOSIS KIT (Ebioscience, USA) and analysed by Flow cytometry according to the instructions (BD Biosciences, USA).

### 2.11 In vivo experiments

RCC1 knockdown was performed by lentiviral vector for RNA interference. The lentivirus particles that short hairpin RNA specific for mouse RCC1 (RCC1 shRNA) or scrambled oligonucleotides (Negative control shRNA) were synthesized by GenePharma (Shanghai, China). The sequences used were as follows: 5′‐GCCGTGTACCTGAATTATT‐3′ for RCC1, and 5′‐TTCTCCGAACGTGTCACGT‐3′ for scrambled sequence. The shRNA‐RCC1 gene fragment was inserted into the lentiviral vector pGLV3/H1/GFP + Puro to construct the lentiviral expression LV3‐RCC1‐shRNA. To construct LLC‐RCC1‐shRNA and LLC‐scrambled‐shRNA stable transfectant, LLC cells were seeded into 6‐well plates with antibiotic‐free medium overnight and transfected with lentivirus at 30%‐50% confluence. After 48 hours' transfection, puromycin (5 μg/mL) was used to treat the cells for 7 days, and knockdown efficiency was assessed by qPCR and Western blot analysis.

Mice were randomly divided into two groups—the NC group and the shRCC1 group—with 8 mice per group, and total 1 × 10^7^ LLC‐RCC1‐shRNA cells or LLC‐scrambled‐shRNA cells were suspended in 100 μL of PBS and injected subcutaneously into the left armpits of 6‐week‐old C57BL/6J wild‐type mice, respectively. After 1 week, when the tumours were measurable, the mice were randomly assigned into 4 groups with 4 mice each (“PD‐L1” in the name of groups represent “PD‐L1 monoclonal Antibody”).

Group I: NC‐shRNA group
Group II: NC‐shRNA group + PD‐L1
Group III: RCC1‐shRNA group
Group IV: RCC1‐shRNA group + PD‐L1

The group II and IV were conducted by intraperitoneal injection (200 μg/mouse in 200 μL PBS saline buffer) every 3 days for a total of 4 injections. Xenograft size was assessed twice a week by calliper measurement and calculated using the following equation: Volume = 0.5 × length × width^2^. The mice were sacrificed in day 22 after injection and the allografts were removed and weighed. Animal experiments were approved by the Ethics Committee of the First Affiliated Hospital of Chongqing Medical University.

### 2.12 Immunohistochemical staining

Tumours allografts were removed and fixed in 4% paraformaldehyde. 4 μm thick paraffin sections were prepared from allografts for immunohistochemistry (IHC) staining. The slides were then deparaffinized with xylene, and rehydrated by using graded ethanol. Antigen retrieval was performed by using microwave heating for 20 minutes in sodium citrate‐hydrochloric acid buffer. Next, the sections were incubated at 4°C overnight with primary antibody: the anti‐RCC1 rabbit antibody (1:500, ABclonal, China), anti‐p27^kip1^ rabbit antibody (1:500, ABclonal, China), anti‐CDK4 rabbit antibody (1:500, ABclonal, China) and anti‐PD‐L1 rabbit antibody (1:500, ABclonal, China). Biotinylated goat anti‐mouse or goat anti‐rabbit antibody was used as a secondary antibody for 20 minutes at room temperature, followed by colour development with DAB. Ultimately, the slides were counterstained with haematoxylin. A light microscope was used to observe and capture the images of histopathological changes.

### 2.13 Statistical analysis

All data were expressed as mean ± standard deviation (SD), and all statistical analyses were performed using the software GraphPad Prism version 7.0. Briefly, the Student's *t*‐test or analysis of variance (ANOVA) was used to analyse the data. *P*‐value of less than .05 was considered statistically significant.

## 3 RESULTS

### 3.1 Up‐regulation of RCC1 correlated with lung cancer

**FIGURE 1:**
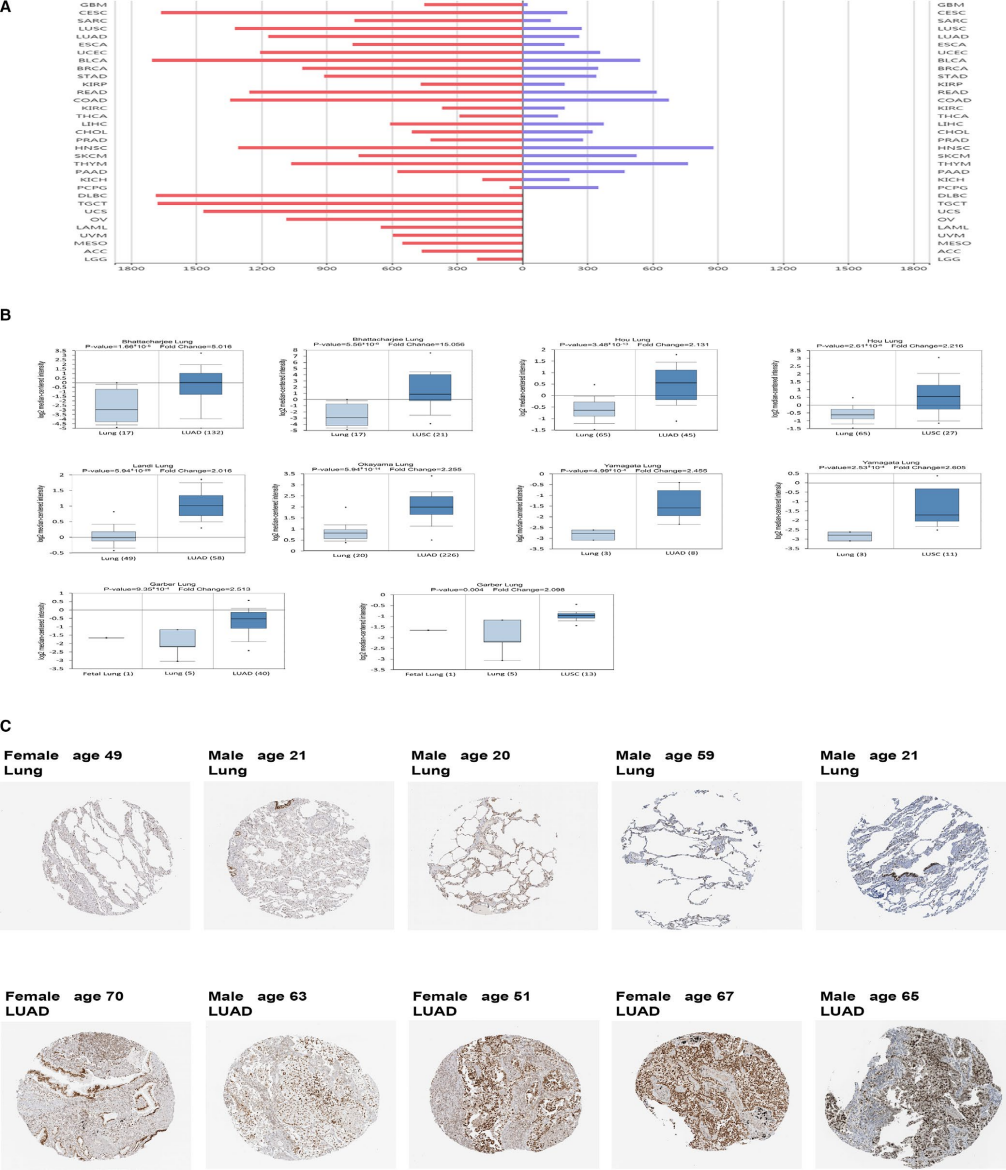
The expression level of RCC1 in lung cancer and normal tissues. (A) The expression level of RCC1 was found in TCGA database. (B) The expression of RCC1 in lung cancer and normal lung tissues was analyzed by Oncomine database. (C) The Human Protein Atlas database was used to detect the immunohistochemical staining of RCC1 in lung cancer tissues and normal lung tissues. Data are shown as the mean ± SD. *P < .05, **P < .01 and ***P < .001

We found the top ten different expression genes (PYCR1, PPP1R14B, ALDH18A1, EFNA4, EPCAM, CNOT11, CCT3, RCC1, PAICS) between lung cancer and paracancerous tissues in TCGA (Table S1). Among them, RCC1 is the only gene which mainly related to cell cycle. In order to understand the relationship between RCC1 and cancer, we elucidated the expression level of RCC1 in different tumour types. It was found that not only in the lung adenocarcinoma and squamous cell carcinoma but also in most other tumour types, the expression level of RCC1 in cancer was significantly higher than that in surrounding tissues (Figure 1A). On the other hand, in Oncomine database, we also proved that the expression of RCC1 in lung cancer was significantly higher than that in normal tissues of lung (Figure 1B and Table S2). Moreover, we confirmed the much higher expression of RCC1 in lung adenocarcinoma in immunohistochemistry database (Figure 1C and Table S3).

### 3.2 RCC1 up‐regulation in lung cancer was correlated with unfavourable patient survival

**FIGURE 2:**
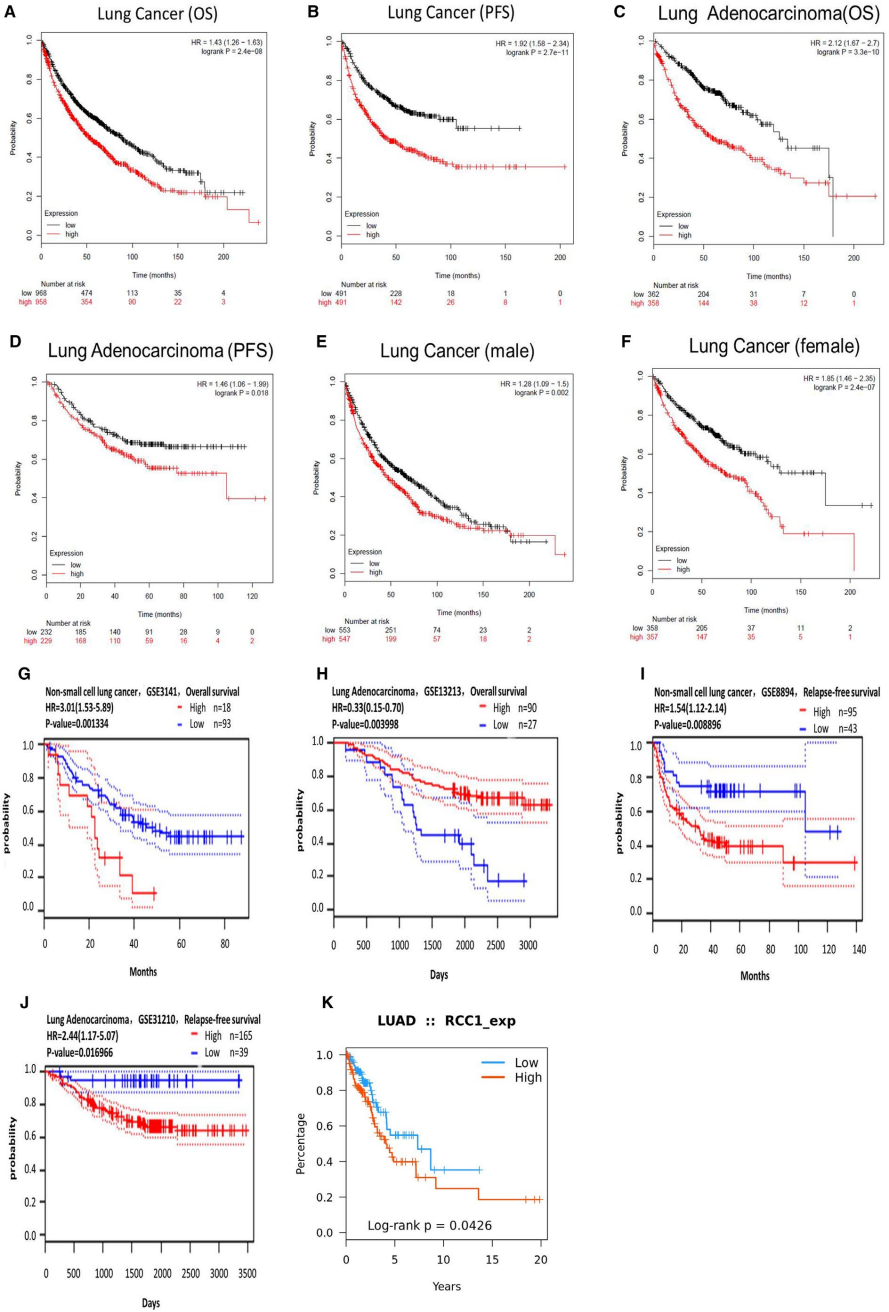
The comparision of the prognostic information between high and low expression of RCC1 from three survival databases. Figure A‐F were from the KM‐plotter database. Figure G‐J were from the Prognoscan database. Figure K was from the TISIDB database.

In Kaplan‐Meier plotter, Prognoscan and TISIDB databases, it was found that higher expression of RCC1 was associated with poor survival and prognosis in lung cancer (Figure 2). These results suggest that high expression of RCC1 in lung cancer might be a potential factor of the carcinogenesis and progress of lung cancer.

### 3.3 RCC1 knockdown inhibited proliferation and induced apoptosis

**FIGURE 3:**
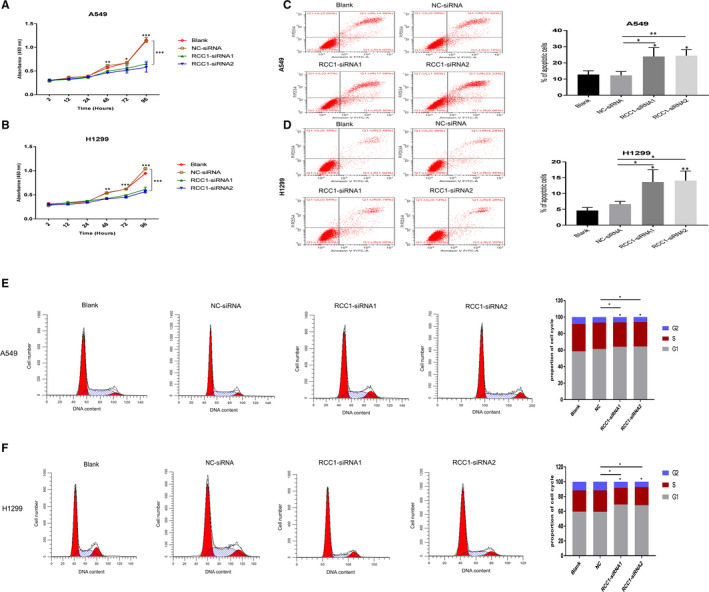
Effect of RCC1 expression on the function of lung adenocarcinoma cells. (A and B) Knockdown of RCC1 expression hindered A549 and H1299 cells growth. (C and D) A549 and H1299 cells were used for flow cytometry analysis assay to measure cell apoptosis. (E and F) A549 and H1299 cells were used for flow cytometry analysis assay to measure cell cycle. Data are shown as the mean ± SD. *P < .05, **P < .01 and ***P < .001

Based on the expression level of RCC1 in lung cancer and the survival analysis from the public database, we next performed experiments to verify the regulation of RCC1 in proliferation, apoptosis and cell cycle. We used siRNA to inhibit the mRNA and protein expression of RCC1 in A549 and H1299 human lung adenocarcinoma cells (Figure S1A,B). It was found that the proliferation rate of the cells was significantly lower than that of the blank and NC group by using CCK8 method (Figure 3A,B). And under the RCC1‐knockdown condition, the apoptosis rates were much higher than other conditions, which were detected by AO/EB staining (Figure 3C,D). Analysis of cell cycle distribution of A549 and H1299 cells showed that knockdown of RCC1 inhibition stalled cells in G1 phase (Figure 3E,F).

### 3.4 Relationship between RCC1 and immune infiltration

**FIGURE 4:**
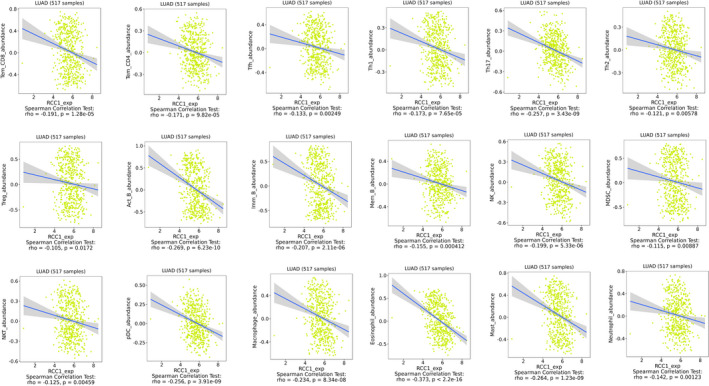
The high expression level of RCC1 was related to the decrease of immune cell infiltration in TISIDB

Next, we confirmed whether RCC1 could influence the immune cells in tumour microenvironments or not. Therefore, the relationship between the expression of RCC1 and the abundance of various immune cells was explored by TISIDB database. We found that the higher expression of RCC1 was related to lower abundance of most immune cells (Figure 4).

### 3.5 Low expression of RCC1 increase the expression of PD‐L1 by regulating p27^kip1^ and CDK4 pathway

**FIGURE 5:**
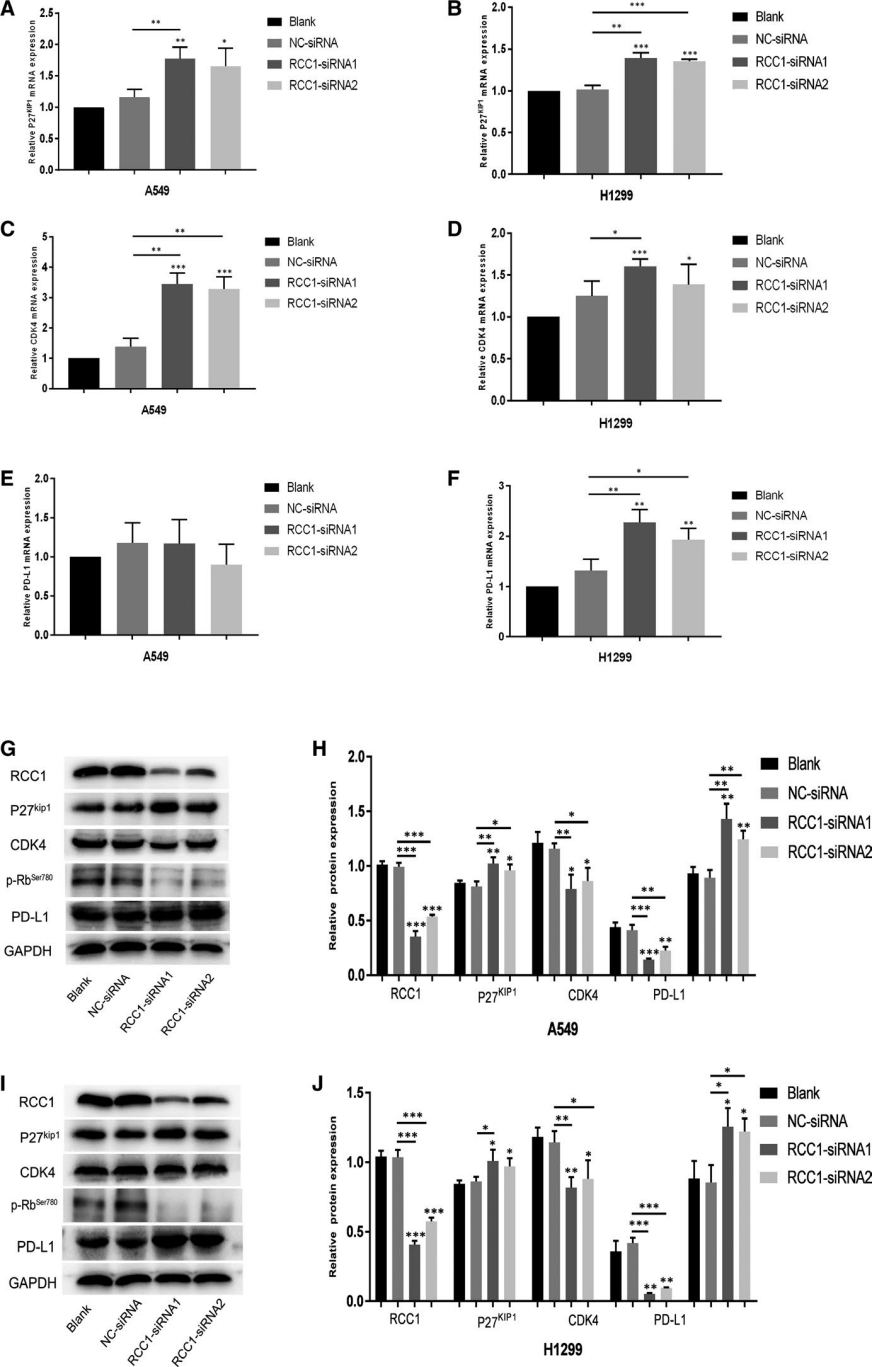
The expression levels of p27^kip1^/CDK4/p‐Rb/PD‐L1 downstreams of RCC1. (A, C and E) The mRNA expression levels of p27^kip1^/CDK4/PD‐L1 in A549 cells detected by qPCR. (B, D and F) The mRNA expression levels of p27^kip1^/CDK4/PD‐L1 in H1299 cells detected by qPCR. (G and H) The protein expression levels of p27^kip1^/CDK4/p‐Rb/PD‐L1 in A549 cells detected by Western blot. (I and J) The protein expression levels of p27^kip1^/CDK4/p‐Rb/PD‐L1 in H1299 cells detected by Western blot. Data are shown as the mean ± SD. *P < .05, **P < .01 and ***P < .001

Moreover, we detected the expression of downstream molecules of RCC1. We found that p27^kip1^ and CDK4 increased in both lung adenocarcinoma cell lines, while PD‐L1 increased only in H1299 cell line at the mRNA level by RCC1 knockdown (Figure 5A‐F). On the other hand, at the protein level, the expression of p27^kip1^, CDK4 and PD‐L1 were changed after knocking downing the expression of RCC1. Low expression of RCC1 significantly increased the protein level of p27^kip1^ and decreased the protein levels of CDK4 and p‐Rb, respectively (Figure 5G‐J). Thus, these results indicated that the depletion of RCC1 might up‐regulate PD‐L1 in lung cancer through inhibiting p27^kip1^/CDK4 axis.

### 3.6 Low expression of RCC1 combined with PD‐L1 antibody inhibited tumour growth

**FIGURE 6:**
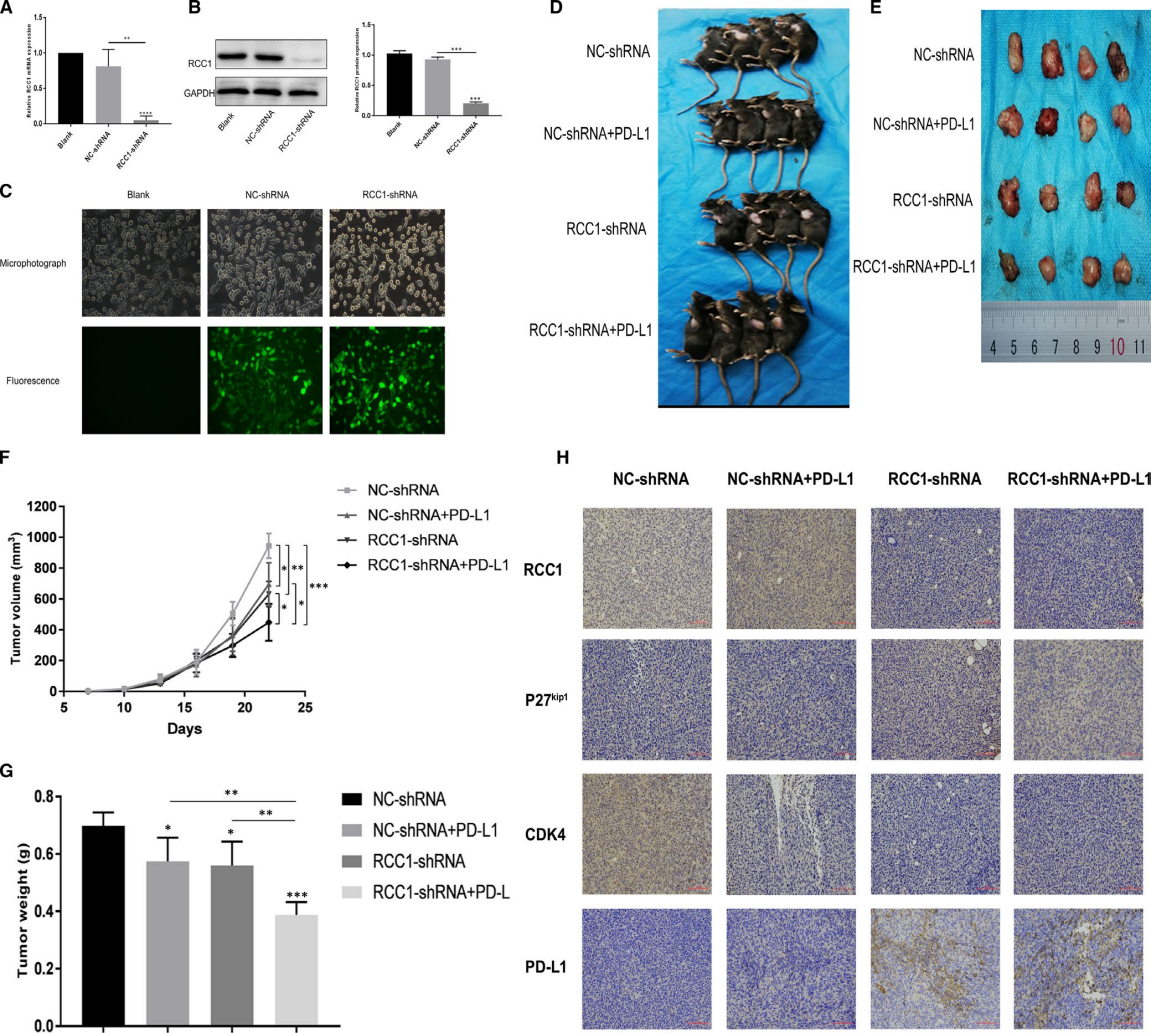
RCC1‐shRNA plus PD‐L1 monoclonal antibody suppressed tumor growth in vivo. (A, B and C) Established a stable RCC1‐shRNA knockdown transfectant‐ RCC1‐shRNA‐Lewis and the negative control line detected by qPCR, Western blot and Fluorescence. (D and E) Image of the tumors before and after we resected. (F and G) Growth curve of tumors volume and tumors weight in 4 groups. (H) Representative images of immunohistochemistry (IHC) staining and stained for RCC1, p27^kip1^, CDK4 and PD‐L1. Data are shown as the mean ± SD. *P < .05, **P < .01 and ***P < .001

**FIGURE 7:**
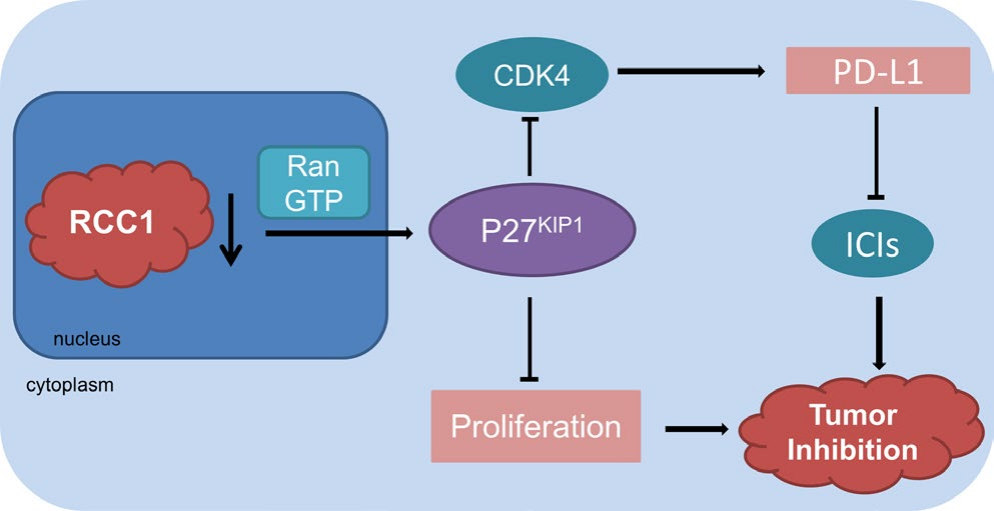
A summary which indicated by in vitro and in vivo results in this study

After down‐regulating the expression of RCC1 at both mRNA and protein levels by lentivirus in Lewis cell line, we established a stable transfectant‐ RCC1‐shRNA‐Lewis and the negative control (Figure 6A‐C). And, to explore the function of RCC1 in vivo, we established tumour models in C57BL/6J mice by transplanting RCC1‐shRNA‐Lewis cells and the negative control cells. We found that the RCC1 knockdown significantly slowed the tumour growth than the negative control group. At the same time, RCC1 knockdown combined with PD‐L1 monoclonal antibody significantly decreased the tumour volume and weight than that of tumours derived from other groups (Figure 6D‐G). The IHC staining indicated that of the low expression of RCC1 tumours related higher PD‐L1 expression (Figure 6H). Figure 7 was a summary which indicated by in vitro and in vivo results in this study (Figure 7).

## 4 DISCUSSION

As a Ras‐related protein, Ran plays a key role in the Ras pathway. It is reported that GTP‐bound Ran is in its functional state in nucleus. And RCC1 contributes to transform RanGDP into RanGTP, and resulting in the transporting of p27^kip1^ outside the nucleus for degradation.^7^, ^18^ Moreover, RCC1 could transfer some other proteins in and out nucleus to regulate the function of CDK4. In this study, we found that the high expression of RCC1 in lung adenocarcinoma might increase the proliferation rate of cancer cells, and related to poor prognosis of patients. On the other hand, down‐regulation of RCC1 expression could inhibit tumour growth both in vitro and in vivo. Therefore, our results suggested that RCC1 should be a hub molecule in the development of lung adenocarcinoma.

High expression of RCC1 could reduce mRNA export from the nucleus, and shut off the synthesis of the proteins required for the G1/S transition.^19^, ^20^ It was reported that PD‐L1 abundance fluctuated in each cell cycle in some cancer cell lines, peaking in M/early G1 phases, and reduction in late G1/S phases.^15^ Therefore, we predicted that RCC1 could regulate the protein level of PD‐L1 because PD‐L1 might be regulated by CDK4 inhibitors through inhibiting the phosphorylation of SPOP. And we performed some experiments to verify it in this study. By inhibiting the expression of RCC1, we elucidated that RCC1 could increase PD‐L1 expression by regulating p27^kip1^/CDK4 axis, which might sensitize the efficacy of PD‐L1 monoclonal antibody. Based on our results, RCC1 could regulate the protein level of downstream molecules. However, it was found that the decrease of RCC1 could also impact the transcriptional level of the downstream genes, which might involve some complex molecular mechanisms. We found the decrease of RCC1 expression linked to the enhancement of CDK4 inhibition and decrease the phosphorylation of Rb, resulting in the decrease of cell number in G1 phase, and the increase of PD‐L1 abundance. Our results indicated that RCC1 could not only inhibit cell growth but also regulate the abundance of PD‐L1 in different cell cycle, which might sensitize the efficacy of immunotherapy.

The microenvironment of tumour is different from that of normal tissue because of the tumour cells and insufficient nutrient supply.^21^, ^22^, ^23^ We found that the high expression of RCC1 is related to the decrease of the number of immune cells. Some kinds of gene alternations, such as EGFR mutation might cause the resistance to ICI therapy, and sometimes even the hyperprogressive disease (HPD).^24^, ^25^ The mechanisms of HPD are not completely clear, which might be associated with the activation of related pathways, and the depletion of immune cells.^26^ High expression of RCC1 leads to the decrease of the number of immune cells might be involved in the induction of HPD. Moreover, if we decrease the expression of RCC1, the depletion of immune cells may be stopped. Therefore, it's worthy for further research.

The suitable biomarkers of immunotherapy are important for selecting suitable cancer patients for ICIs treatment. Currently, both PD‐L1 and tumour mutation burden (TMB) are prognostic markers for ICIs.^27^ As a ligand of PD‐1, PD‐L1 is a transmembrane protein on the cell membrane, expressed in T cells, B cells and tumour cells.^28^, ^29^, ^30^ It is judged as positive when the tumour Proportion Score (TPS) > 1%.^31^, ^32^ The relationship of PD‐L1 expression level and ICIs resistance has aroused our interest. Atezolizumab is a commercialized PD‐L1 antibody, which could be applicated in advanced NSCLC patients. The efficacy of PD‐L1 positive patients is better than those without expression of PD‐L1.^33^ However, the clinical trials of Checkmate 017 and Checkmate 057 suggested that the efficacy of Nivolumab was better than chemotherapy even in PD‐L1 negative patients.^34^, ^35^ TMB is a complex marker because of high price, and no uniform standard testing methods. Moreover, although the TMB of some patients is very high, the effectiveness of ICI is still poor.^36^, ^37^ Therefore, if RCC1 could be viewed as a marker, the combination of RCC1 with PD‐L1 or TMB might play an important role in screening the dominant population for ICIs treatment.

It was reported that the 3‐year overall survival of the PACIFIC study was 56%, and the estimated 5‐year OS might around 40%‐50%, which indicated the best result compare to previous research.^38^ Thus, several guidelines have adopted Durvalumab maintenance therapy as the standard treatment for locally advanced NSCLC after concurrent chemoradiotherapy (CCRT).^39^, ^40^ Inhibition of the increased PD‐L1 level after chemoradiotherapy might increase tumour immunity and prolong PFS.^41^, ^42^ Thus, ICIs could be used as maintenance therapy after CCRT for patients with low expression of RCC1 and high expression of PD‐L1. On the other hand, it might be meaningless to the patients with high expression of RCC1 and low expression of PD‐L1 after CCRT. The results of Pacific study also indicated that low expression of PD‐L1 related to shorter PFS compared with high expression of PD‐L1. Therefore, it is helpful to further select the suitable patients.

In general, inhibition of RCC1 expression might not only inhibit the development of lung adenocarcinoma but also increase the protein level of PD‐L1, which might sensitize the efficacy of PD‐L1 monoclonal antibody. These results suggested that in clinical practice, the combination of ICIs and RCC1 inhibitor might achieve more satisfactory results.

## CONFLICT OF INTEREST

The authors declare no conflict of interest.

## AUTHOR CONTRIBUTIONS

**Xiaozhu Zeng:** Conceptualization (equal); data curation (equal); methodology (equal); software (equal); writing‐original draft (equal). **Maoxi Zhong:** Data curation (equal); formal analysis (equal); methodology (equal). **Yumeng Yang:** Software (equal); writing‐original draft (equal). **Zhi Wang:** Methodology (equal); software (equal). **Yuxi Zhu:** Conceptualization (equal); project administration (equal); writing‐review & editing (equal).

## Supporting information

Supplementary Material

## Data Availability

The data that support the findings of this study are available from the corresponding author upon reasonable request.
