# Supplementary material for: Down‐regulation of RCC1 sensitizes immunotherapy by up‐regulating PD‐L1 via p27kip1/CDK4 axis in non‐small cell lung cancer
Source: J Cell Mol Med. 2021 Feb 25;25(8):4136–47. doi: 10.1111/jcmm.16383 (PMC8051708; doi:10.1111/jcmm.16383)
Supplement: Supplementary file 1 — Supplementary Material [file JCMM-25-4136-s001.docx]

Supplementary Materials for

**Down-regulation of RCC1 sensitize immunotherapy by upregulating PD-L1 via p27kip / CDK4 axis in Non-small cell lung cancer**

Xiaozhu Zeng, Maoxi Zhong, Yumeng Yang, Zhi Wang, Yuxi Zhu

Correspondence to: zhuyuxi@hospital.cqmu.edu.cn

**This PDF file includes:**

Supplemental Figures and Tables

Figures S1

Tables S1 to S4


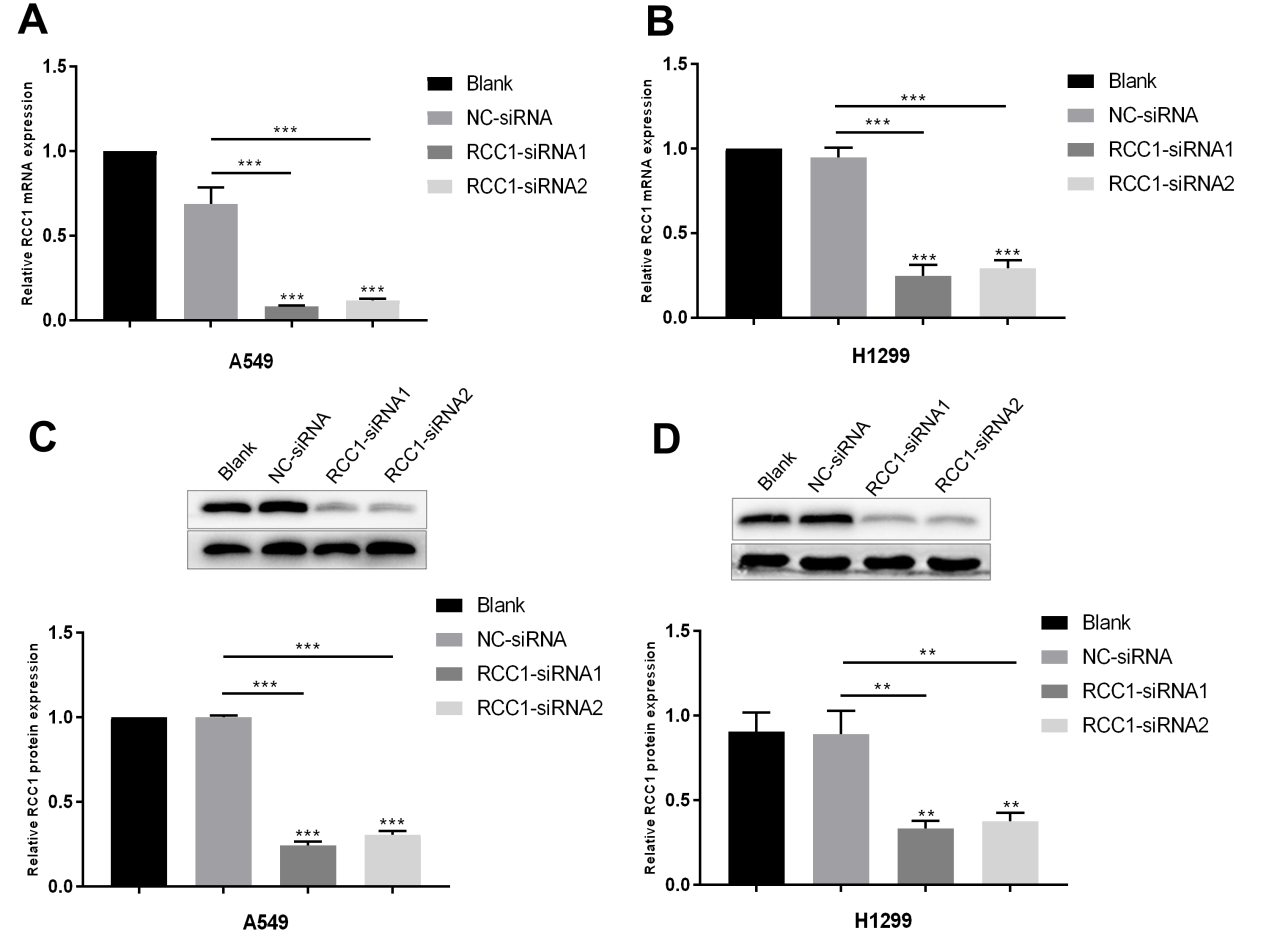


Supplementary Figure S1. Knockdowning the mRNA and protein expression of RCC1 in A549 and H1299 cells detected by mRNA and protein.

Supplementary Table S1. The first ten differentially expressed genes.

| Gene | Log(P-value) | LogFC |
| --- | --- | --- |
| PYCR1 | -119.178 | 6.803695 |
| PPP1R14B | -111.482 | 6.977637 |
| ALDH18A1 | -110.109 | 5.889948 |
| EFNA4 | -101.801 | 5.190837 |
| EPCAM | -99.5893 | 8.520354 |
| CNOT11 | -99.3762 | 5.533258 |
| CCT3 | -98.9035 | 7.202907 |
| RCC1 | -98.7793 | 5.148323 |
| PAICS | -97.6102 | 5.246947 |

Supplementary Table S2. Up-and down-regulated expressions of RCC1 in the studies identified in the Oncomine database.

| Type of cancer | Up-regulated  (No．of studies) | Down-regulated (No. of studies) |
| --- | --- | --- |
| Bladder cancer | 4 | 0 |
| Brain and CNS cancer | 7 | 1 |
| Breast cancer | 5 | 0 |
| Cervical cancer | 0 | 0 |
| Colorectal cancer | 4 | 0 |
| Esophageal cancer | 1 | 0 |
| Gastric cancer | 1 | 0 |
| Head and neck cancer | 2 | 0 |
| Kidney cancer | 0 | 0 |
| Leukemia | 4 | 3 |
| Liver cancer | 0 | 0 |
| Lung cancer | 13 | 0 |
| Lymphoma | 2 | 0 |
| Melanoma | 1 | 0 |
| Myeloma | 1 | 0 |
| Other cancers | 2 | 0 |
| Ovarian cancer | 1 | 0 |
| Pancreatic cancer | 0 | 0 |
| Prostate cancer | 3 | 0 |
| Sarcoma | 2 | 0 |

CNS: central nervous system.

Supplementary Table S3. Detailed information about IHC results of RCC1.

|  | Gender | Age | Antibody | Antibody Staining | Intensity | Quantity |
| --- | --- | --- | --- | --- | --- | --- |
| Normal | Female | 49 | HPA027573 | Medium | Moderate | >75% |
|  | Male | 21 | HPA027573 | Medium | Moderate | >75% |
|  | Male | 20 | HPA027574 | Medium | Moderate | >75% |
|  | Male | 59 | CAB015413 | Medium | Moderate | 75%-25% |
|  | Male | 21 | CAB015413 | Medium | Moderate | 75%-25% |
| Tumor | Female | 70 | HPA027573 | High | Strong | >75% |
|  | Male | 63 | HPA027574 | High | Strong | 75%-25% |
|  | Female | 51 | HPA027573 | High | Strong | >75% |
|  | Female | 67 | HPA027574 | High | Strong | >75% |
|  | Male | 65 | CAB015413 | High | Strong | >75% |

Supplementary Table S4. The sequence of primers used in qPCR.

| Gene | Species | Forward primer (5′-3′) | Reverse primer (5'-3') |
| --- | --- | --- | --- |
| RCC1 | homo | CGGCCCTGGTATCCATTCC | CACTTTTGCTTAGACACACGGT |
|  | mus | ACACAGGTCCCACAACACAG | GCCTTCGACTGAAGTGTCCC |
| P27^KIP1^ | homo | AGGAGGAGATAGAAGCGCAGA | GTGCGGACTTGGTACAGGT |
| CDK4 | homo | ATGGCTACCTCTCGATATGAGC | CATTGGGGACTCTCACACTCT |
| PD-L1 | homo | TGGCATTTGCTGAACGCATTT | TGCAGCCAGGTCTAATTGTTTT |
| GAPDH | homo | TCAAGAAGGTGGTGAAGCAGG | AGCGTCAAAGGTGGAGGAGTG |
|  | mus | AGGTCGGTGTGAACGGATTTG | GGGGTCGTTGATGGCAACA |
